# Discovery Beyond the “Undiscovered Country”: Re-Exploring the Right Ventricle Through Metabolomics and Cardiac Magnetic Resonance

**DOI:** 10.1016/j.jacasi.2025.12.002

**Published:** 2025-08-29

**Authors:** Yue-Hin Loke

**Affiliations:** Department of Pediatric Cardiology, Children’s National Hospital, Washington, DC, USA

**Keywords:** metabolomics, pressure overload, pulmonary arterial hypertension, repaired tetralogy of Fallot, volume overload

**Figure:**
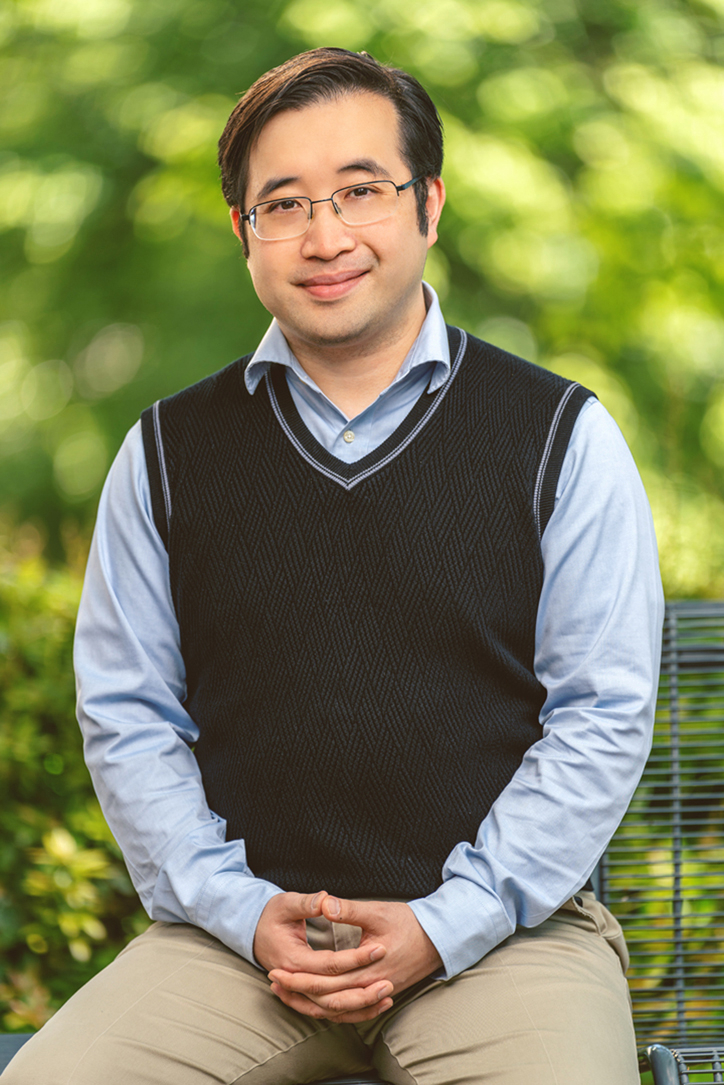

“But that the dread of something after death, The undiscover’d country from whose bourn No traveller returns, puzzles the will And makes us rather bear those ills we have Than fly to others that we know not of?”—Hamlet, Act III, Scene I^1^

For much of our understanding in cardiovascular medicine, the right ventricle (RV) was considered a “forgotten chamber” despite its central role in congenital and pulmonary vascular disease.^2^ Maladaptation of RV to abnormal loading is best characterized by cardiac magnetic resonance (CMR). CMR parameters such as RV size, ejection fraction, and scar burden have been rigorously validated.^3^, ^4^, ^5^, ^6^ Yet, these parameters have largely been calibrated toward determining the risk of mortality, the “undiscovered country.”^7^ Equally essential, yet daunting and complex, is establishing the link with functional capacity to better capture the lived experiences and quality of life of our patients.

Against this backdrop, in this issue of *JACC: Asia*, Zhao et al^8^ report an interesting study integrating CMR, cardiopulmonary exercise testing, and metabolomic profiling in 2 distinct models of RV stress: repaired tetralogy of Fallot (rTOF) as a form of chronic volume overload and pulmonary arterial hypertension (PAH) representing pressure overload.

In a prospective cohort of 227 subjects (103 control subjects, 67 rTOF patients, 57 PAH patients), participants underwent multiparametric CMR, cardiopulmonary exercise testing, and targeted metabolomics analysis. rTOF patients demonstrated greater RV dilatation, whereas PAH patients exhibited worse exercise tolerance and more advanced functional impairment (eg, lower peak oxygen uptake). Metabolically, rTOF was characterized by elevations in branched-chain amino acids (BCAAs), which correlated negatively with CMR-derived RV function including tricuspid annular plane systolic excursion and RV global longitudinal strain. In contrast, PAH was marked by elevations in acyl-carnitines and one-carbon metabolites such as dimethylglycine and choline, consistent with mitochondrial/oxidative stress. These biomarkers were also associated with adverse remodeling (increased RV mass and volume) and impaired function (reduced right ventricular ejection fraction [RVEF]). Notably, receiver-operating characteristic (ROC) analyses demonstrated that select metabolites were noninferior in prediction of exercise intolerance in both rTOF and PAH when compared with RVEF.^8^

Zhao et al’s work highlights that pressure and volume loading produce not only divergent structural and functional remodeling, but also distinct circulating metabolic signatures, offering new opportunities for mechanistic insight and future clinical application. These findings build on prior work showing that conventional volumetric thresholds, such as RV end-diastolic volume or pulmonary regurgitant fraction, are imperfect predictors of functional decline,^9^^,^^10^ and that outcomes are more tightly linked to late-stage dysfunction than chamber size alone.^11^^,^^12^ Complementary CMR 4-dimensional (4D) flow studies have further revealed that both rTOF and PAH are characterized by abnormal intraventricular flow environments that correlate with impaired exercise tolerance.^13^^,^^14^ Together, these studies suggest that the maladaptive RV in rTOF and PAH cannot be reduced to “volume overload” or “pressure overload” as captured by conventional imaging indices, and rather reflects a complex interplay of remodeling, energetics, and metabolic stress. Importantly, the metabolomics perspective is in line with investigations of broader congenital heart disease cohorts, in which larger profiling studies implicate pathways like arginine biosynthesis in adverse RV phenotypes.^15^ In this context, Zhao et al’s demonstration of metabolomics adds novel and disease-specific dimensions to our understanding of maladaptive remodeling.

The elevation of circulating BCAAs observed in rTOF may reflect both cardiac dysfunction and broader systemic disturbances. Prior 4D flow CMR studies by Zhao et al and others for rTOF have demonstrated reduced direct flow, diminished systolic kinetic energy, and elevated viscous energy loss as flow abnormalities that contribute to RV mechanical inefficiency and maladaptive remodeling.^13^^,^^16^ These alterations in hemodynamics likely indirectly affect BCAA substrate utilization, as outlined by McGarrah et al.^17^ BCAA metabolism is a distributed process, with the majority of oxidation occurring in skeletal muscle, liver, and adipose tissue, while the heart contributes only a minor share. In cardiovascular disease states, including heart failure, transcriptional downregulation of key enzymes impairs cardiac BCAA catabolism, leading to accumulation of BCAAs and their α-ketoacid derivatives. These metabolites exert multiple pathogenic effects: activation of the mTOR pathway (which impairs autophagy and promotes insulin resistance), inhibition of mitochondrial complex I (leading to superoxide production and oxidative stress), and suppression of glucose oxidation (favoring lipid accumulation and metabolic inflexibility). Notably, BCAA catabolism can be activated pharmacologically, and dietary restriction of BCAAs may also improve metabolic health and reduce frailty in animal models.^17^, ^18^, ^19^ Therefore, BCAA dysregulation in rTOF may serve not only as a biomarker of disease burden, but also as a potential therapeutic target for RV maladaptation.

For PAH, Zhao et al demonstrated through 4D flow CMR that the RV is also subject to abnormal hemodynamic forces, resulting in reduced direct inflow and higher residual volumes that were also independently predictive of impaired functional capacity. These flow properties likely reflect inefficient intraventricular energy transfer and increased myocardial energetic burden.^14^ Within this context, the observed elevation of one-carbon metabolites including dimethylglycine, choline, and S-adenosylhomocysteine likely represents a biochemical footprint of pressure-induced metabolic strain. These metabolites are tightly linked to mitochondrial redox balance and methylation capacity, and their accumulation suggests impaired oxidative metabolism, elevated reactive oxygen species, and disrupted epigenetic regulation. As reviewed by Koh et al,^20^ mitochondrial oxidative stress is a defining feature of cardiovascular aging and maladaptive remodeling, promoting DNA damage, lipid peroxidation, and endothelial dysfunction. In the pulmonary vasculature, redox stress impairs nitric oxide signaling and contributes to increased vascular stiffness and resistance, which are also key drivers of PAH pathophysiology. Together, these flow and metabolite findings support a model in which chronic RV pressure overload initiates a cascade of hemodynamic inefficiency and redox imbalance, with one-carbon metabolites serving as measurable intermediaries of this maladaptive state.

Several important caveats temper the interpretation of these findings. While rTOF is a common and clinically relevant model of RV dilation, it is not a pure volume overload state; residual pulmonary regurgitation frequently coexists with restrictive physiology, myocardial fibrosis, and conduction abnormalities.^5^ In some patients, the observed chamber enlargement may be driven less by regurgitant load and more by electromechanical dyssynchrony, distinguishing it from volume-loading phenotypes such as atrial septal defects or tricuspid insufficiency. From a biochemical perspective, the use of peripheral venous sampling for metabolomic profiling limits cardiac-specific interpretations, as systemic metabolite concentrations reflect integrated signals from multiple organs (ie, skeletal muscle, liver, adipose tissue, and kidney), not just the heart. As others have emphasized, the heart accounts for only a small fraction of overall BCAAs and one-carbon metabolism, and without myocardial-specific sampling or spatially resolved imaging-biochemistry correlation, tissue of origin remains speculative.^17^^,^^21^ Similarly, while Zhao et al report that select metabolites demonstrate noninferior predictive performance compared with RVEF for exercise intolerance, these ROC analyses should be interpreted cautiously. RVEF itself has known limitations in RV pathophysiology, especially in diseases with regional dysfunction, altered loading conditions, or uncoupled flow-volume relationships.^22^ The results are interesting in the context of an exploratory, hypothesis-generating study, but they emphasize the need for careful validation in larger, outcomes-driven datasets. Finally, while the metabolomic patterns observed offer compelling insight into underlying biology, the translational significance remains speculative. Whether metabolic interventions, such as modulating BCAA catabolism, can improve outcomes in congenital or pulmonary vascular disease is unknown. These findings should therefore be viewed as an important foundation for mechanistic understanding, but future work will need to link these molecular phenotypes to causal pathways and actionable clinical strategies through interventional or longitudinal studies.

Despite these limitations, the study advances several important themes. Traditional metrics such as RV mass, ejection fraction, and strain fail to fully predict exercise intolerance in PAH or rTOF. The present findings suggest that metabolic changes may capture alternative dimensions of RV maladaptation. Measuring these metabolite levels could also be implemented in longitudinal follow-up of patients and guide the timing of afterload-reducing strategies in PAH or surgical and valve-based interventions in rTOF. The clinical context is particularly compelling for rTOF: transcatheter pulmonary valve replacement has expanded dramatically, with over 1,200 annual implantations,^23^ yet decisions on timing still hinge on volumetrics that incompletely capture disease burden.

Looking ahead, future research should move beyond cross-sectional associations toward longitudinal, mechanistically grounded studies. Large, multicenter cohorts with standardized imaging and metabolomic protocols are essential to validate these findings and determine whether specific metabolic signatures in rTOF or PAH can predict clinical outcomes beyond exercise intolerance, including disease progression, response to therapy, or adverse events. A key next step will be to perform serial metabolic profiling linked to defined clinical intervention points, such as the timing of pulmonary valve replacement in rTOF or initiation of advanced therapy in PAH. This approach could help determine whether changes in metabolite levels precede or follow structural or functional decompensation, informing optimal intervention timing. Just as CMR protocols have been harmonized across imaging core labs, metabolomic assay standardization, including sample collection, storage, batch correction, and analyte quantification, must be prioritized to ensure reproducibility and facilitate cross-site validation.^20^^,^^24^ In parallel, the integration of metabolomic data with multiparametric imaging and exercise testing into predictive risk models may offer a more comprehensive, systems-level approach to stratifying RV disease severity. Importantly, the heterogeneity within PAH and rTOF cohorts argues against one-size-fits-all solutions; future studies should embrace this complexity and evaluate whether metabolomics can help disentangle biologically meaningful subgroups within these overlapping yet distinct RV pathophenotypes.

Zhao et al provide an important contribution by demonstrating that RV pressure and volume overload produce not only structural/functional remodeling but also divergent circulating metabolic signatures. Their findings underscore the potential of metabolomics to complement imaging in understanding and predicting RV dysfunction. The work is exploratory, but points to a future where the integration of imaging, exercise testing, and biochemical phenotyping may allow us to move beyond descriptive metrics toward mechanism-based risk stratification and therapy. For now, the study should be viewed as a critical step forward, inviting a more holistic approach to RV disease. This effort reflects a journey beyond the “undiscovered country” of the RV, in which clinical management is not only “to be or not to be” in terms of survival, but also enabling patients to thrive as cardiologists boldly go toward a richer understanding of patient experience.

## Funding Support and Author Disclosures

The authors have reported that they have no relationships relevant to the contents of this paper to disclose.
